# Train vs. Play: Evaluating the Effects of Gamified and Non-Gamified Wheelchair Skills Training Using Virtual Reality

**DOI:** 10.3390/bioengineering10111269

**Published:** 2023-10-31

**Authors:** Chantal Zorzi, Luma Tabbaa, Alexandra Covaci, Konstantinos Sirlantzis, Gianluca Marcelli

**Affiliations:** 1 School of Engineering, University of Kent, Canterbury CT1 7NT, UK; cz77@kent.ac.uk (C.Z.); l.a.tabbaa@kent.ac.uk (L.T.); a.covaci@kent.ac.uk (A.C.); 2 School of Engineering, Technology and Design, Canterbury Christ Church University (CCCU), Canterbury CT1 1QU, UK; konstantinos.sirlantzis@canterbury.ac.uk

**Keywords:** virtual reality, wheelchair, training, sensors, gamification, assistive technologies, rehabilitation

## Abstract

This study compares the influence of a gamified and a non-gamified virtual reality (VR) environment on wheelchair skills training. In specific, the study explores the integration of gamification elements and their influence on wheelchair driving performance in VR-based training. Twenty-two non-disabled participants volunteered for the study, of whom eleven undertook the gamified VR training, and eleven engaged in the non-gamified VR training. To measure the efficacy of the VR-based wheelchair skills training, we captured the heart rate (HR), number of joystick movements, completion time, and number of collisions. In addition, an adapted version of the Wheelchair Skills Training Program Questionnaire (WSTP-Q), the Igroup Presence Questionnaire (IPQ), and the Simulator Sickness Questionnaire (SSQ) questionnaires were administered after the VR training. The results showed no differences in wheelchair driving performance, the level of involvement, or the ratings of presence between the two environments. In contrast, the perceived cybersickness was statistically higher for the group of participants who trained in the non-gamified VR environment. Remarkably, heightened cybersickness symptoms aligned with increased HR, suggesting physiological connections. As such, while direct gamification effects on the efficacy of VR-based wheelchair skills training were not statistically significant, its potential to amplify user engagement and reduce cybersickness is evident.

## 1. Introduction

It is estimated that 1% of the global population are wheelchair users (WUs) [1]. To promote the integration of WUs in society and enhance their independence, it is important that they know how to manoeuvre a wheelchair safely [2]. Several training programmes for wheelchair driving skills have been developed; some programmes entail navigating in everyday settings (i.e., homes, schools, etc.), while others focus on controlled environments where a set of tasks are performed [3]. Unfortunately, due to high costs and restricted accessibility, these training programmes are not accessible to many [2,4]. 

In efforts to overcome these barriers to wheelchair training, many researchers have investigated the use of technology to provide wheelchair training that can be low-cost and more accessible [2,3,5]. For instance, research shows that WU training using virtual reality (VR) can increase engagement with tasks [6], can be motivating, and can mitigate the risks faced in real-world training (i.e., risk of falling) [7]. However, these training methods vary widely in nature, including the following three key aspects: the hardware adopted to present the VR environment to the user, the controller for navigation, and the design characteristics of the VR environment itself. This sparks the question of what are the characteristics of an effective VR training system that enables trainees to transfer their learnt skills to real life. To answer this question, each of the mentioned aspects needs to be examined. 

Firstly, the presentation hardware can vary in the level of immersion depending on how much the screen can surround the user to provide a non-immersive (using monitor screens), a semi-immersive (using CAVE systems; a system that projects the virtual environments on walls), or a fully immersive (using head-mounted displays (HMDs)) experience. For instance, John et al. [8] carried out a VR training study for powered wheelchair users with participants split into a control group (no training received), an HMD training group, and a monitor screen training group. The study found an overall higher improvement in skills for the HMD training group, which reduced distractions from the trainees’ surroundings and increased focus on the training. From a user’s experience perspective, Débora et al. [9] found that when participants used HMDs, it elicited higher levels of presence and pleasant and exciting emotions. Increased presence was also found by Alshaer et al. [10], whose results demonstrated that HMDs led to better involvement and ability to navigate through obstacles. These findings suggest that HMDs may offer advantages in wheelchair skills training over other hardware options. 

Secondly, the chosen controller for navigation may depend on the mobility needs of the WUs which can range from joysticks to brain–computer interfaces (BCIs) [11]. As such, VR can be controlled using gaming joysticks [12], powered wheelchair joysticks [6,8,13,14], sensors on the wheels [15], or eye-tracking devices [13], just to name a few. Most of the work in the field of VR for wheelchair skills training focused on powered WUs who use a joystick for navigation in real life [2]. However, joysticks vary from one another, and many VR training programmes use joysticks different from the ones WUs use in their day-to-day life. Undergoing VR training using a different joystick may result in the user having to get adjusted to multiple controllers which vary in mechanical aspects and thus affect the retention of skills. To provide a solution to this issue, Zorzi et al. [16] designed a method of navigation using an inertial measurement unit (IMU) sensor that can be retrofitted to various joysticks. This method has the potential to allow users to train independently and in the comfort of their own chair, making it an efficient alternative to other controllers. 

Thirdly, the design characteristics of the VR environment also varied among the body of literature. Some environments were realistic, recreating a virtual replica of laboratory rooms [13] or rehabilitation centres [6], while others were less realistic and contained elements of gamification, such as having the user collect balls of a specific colour while avoiding collision with other coloured balls [8]. Zorzi et al. [16] proposed a VR environment replicating the (real-life) Wheelchair Skills Training Program (WSTP) [17,18]; they found that VR training for harder tasks enhanced the acquisition of skills in real life. However, their study reported a limitation in their work, mentioning that participants found the environment to be very realistic and disengaging. Adding elements of gamification could be a solution to increase engagement. 

To our knowledge, no study has compared the effects of a non-gamified environment to a gamified environment when it comes to rehabilitation, specifically wheelchair driving skills training. When examining the aesthetics and gamification of the virtual experience and its effects on user performance and retention of skills for the real world, the literature in the context of wheelchair training is lacking. However, there exists strong support in the literature in other contexts of training, which highlights the strength and efficacy of gamification in VR training to further enhance users’ outcomes. For instance, Palmas et al. [19] compared gamified and non-gamified VR musical instrument assembly training and found that the use of gamification can enhance the efficacy of a VR training programme.

Despite the potential of gamification in enhancing trainees’ outcomes in other disciplines, it is unclear whether such effects could be replicated in the context of wheelchair skills training. As such, further research work is needed to investigate whether the gamification of VR training can enhance wheelchair skills training outcomes. This prompts our research question: *Can elements of gamification enhance the VR-based wheelchair training effectiveness?*

We aim to answer the above research question by comparing the acquisition of skills with real-life WSTP tasks, following wheelchair skills training in a “non-gamified” VR environment versus a “gamified” one; this allows one to establish if training in either VR environment leads to better acquisition of skills that are transferable to real life. We believe that the effectiveness of VR training also depends on feelings elicited by the VR environment, specifically presence, involvement, and most importantly cybersickness. Therefore, we consider those aspects when addressing our research question. 

We investigate our research question through the following three hypotheses:

**H_1_.**  *Real-life driving performance after VR training will differ based on the environment.*

To examine potential variations in learning outcomes due to the differences in environment design, we assess post-VR training driving performance. This will be measured with the number of collisions, the completion time per task, the total number of joystick movements per task and through a modified version of the WSTP questionnaire [20].

**H_2_.**  *The levels of involvement and presence will vary based on the environment.*

The sense of presence is believed to increase the effectiveness of VR-based training programmes [5]. Hence, we are interested in seeing if the differences in environmental design affect the sense of presence. This will be measured using the Igroup Presence Questionnaire (IPQ) [21].

**H_3_.**  *The perceived cybersickness will vary based on the environment.*

Cybersickness is one of the main limitations of VR due to the discomfort it brings to the user, including visual fatigue, nausea, disorientation [22], and headaches, which lead to less enjoyment of the VR experience [23]. Hence, we are interested in seeing if the differences in environmental design may help to reduce it. This will be assessed by using the Simulator Sickness Questionnaire (SSQ) [24] and by measuring the heart rate (HR) of the participants (HR is known to change as a result of cybersickness [25]).

The structure of the paper is as follows: Section 2 discusses previous related work and how our study builds on it. Section 3 describes the materials and methods of our study. Section 4 presents the results, which are discussed in Section 5. Finally, the paper ends with our conclusions and future work (Section 6).

## 2. Related Work

In this section, we investigated the different VR environments that have been designed for wheelchair skills training (Section 2.1) and how elements of gamification have been applied in VR training systems (Section 2.2).

### 2.1. Environment Design of Wheelchair Skills Training in VR

Wheelchair skills training using VR has been a topic of research since the 1990s [2]. VR offers a potential solution to the challenges posed by traditional training methods, which require time and resources that may not be accessible to everyone [2]. As such, VR can be a useful supplement to traditional training methods. One of the shortcomings of current VR training approaches is the heterogeneity in their design [2]. Designs vary from realistic replicas of real-life environments such as laboratory rooms [13] or rehabilitation centres [26], while others are less realistic [27].

The body of research highlighted the benefits of using realistic virtual environments and found that they meet the participants’ expectations of real-life powered wheelchair driving [26]. Moreover, participants may easily transfer the skills learnt in VR to real life, given that the tasks in the two contexts are similar [28]. In a study conducted by Torkia et al. [26], participants (clinicians and children who are wheelchair users) were asked for feedback about their VR training experience (driving through a replica of a rehabilitation centre); they suggested increasing the interaction with the VR environment and adding sounds to improve the sense of presence and training efficacy [26]. To achieve these two goals, aspects of gamification could be added to the VR training to enhance the motivation and engagement of the participants [29]. In fact, when participants were asked about the most enjoyable part of their training, they described actions and interactions they had with the virtual world [26]. Furthermore, less realistic environments can resemble gamified worlds, which may be necessary if participants have, for example, multiple disabilities and therefore require specific designs [27]. John et al. [8] used a less realistic environment design that contained gamified tasks and showed that driving skills acquired in VR are retained in real life. Yet, using gamified environments is a less common approach to wheelchair skills training, and so its benefits are not yet fully explored.

### 2.2. Gamification in Training

The term gamification refers to the use of game aspects in non-gaming contexts [30]. Aparicio et al. [31] defined a framework for gamification, which can help improve the participation and motivation of carrying out certain tasks. The authors suggested ways to motivate people using game mechanics (e.g., points, levels, leaderboards, etc.) that favour autonomy, competence, and relatedness.

When examining if game mechanics are effective in VR rehabilitation systems, Kern et al. [32] developed an application to increase motivation by promoting relatedness, autonomy, and competence during gait rehabilitation. In their system, they used elements of gamification, including an engaging storyline, a gamified reward system, and a social companion. Compared to traditional rehabilitation, their system allowed for increased decision freedom, increased perceived task meaningfulness, lower anxiety, lower frustration, and lower physical demand. 

Gamification was also found useful by Putze et al. [33], who developed gamified training for motor imagery brain–computer interfaces (MI-BCIs). The elements of gamification included were a themed environment, score points, progressive increase in speed across several training runs, and levels. The results showed that gamification improves MI-BCI skills for beginner users and stimulates low levels of fatigue. 

The use of game mechanics in VR training has also been found effective in the improvement of skills in real life by Ulmer et al. [34] in training for assembly tasks. The authors [34] suggested that even though gamified VR could provide support for the completion of the tasks at the beginning of training, this positive effect can decrease gradually. Furthermore, both positive and negative feedback should be provided throughout the training to balance the participant’s feeling of competence without adding pressure. The authors suggested that effective gamified VR training should motivate the participants to build, retain, and recall their knowledge of the performed tasks.

As for how the users feel about gamified VR training, Yan et al. [35] investigated the acceptance of gamification by older adults. They used a total of six games designed for life-skills training, leisure (e.g., hobbies), and motor exercise development. The findings revealed that after exposure to the VR games, participants felt they were useful and easy to use.

However, the studies directly comparing the effects of gamified VR training to non-gamified ones are very limited. Palmas et al. [19] investigated gamification’s benefits over non-gamification in the context of training assembly tasks and found that it enhanced the efficacy of the VR training application. Despite the existing body of research exploring the benefits of gamification in various contexts, there remains a gap in understanding the specific advantages of using a gamified approach versus a non-gamified one in the domain of rehabilitation, specifically wheelchair driving skills training. The unique challenges and goals of wheelchair skills training necessitate a tailored investigation into the potential benefits of incorporating gamification in VR training systems. 

## 3. Materials and Methods

In this study, we developed two VR environments to conduct wheelchair skills training, using Autodesk Maya [36] to produce all 3D elements and Unity 3D [37] to develop the training system. One environment was designed to be non-gamified (see Section 3.2.2), and one environment was designed to be gamified (see Section 3.2.3). The participant’s performance before and after VR training was tested in real life (see Section 3.2.1). The following subsections discuss participant requirements and ethics (Section 3.1), the setup of the study (Section 3.2), the hardware used in the system (Section 3.3), and the data collected (Section 3.4).

### 3.1. Participants and Ethics

Participants were recruited through the University of Kent body of students and staff. The study was advertised through an email to all the divisions of the university, and interested participants were screened according to the following eligibility requirements: to be over 18 years of age, speak and write fluent English, have little to no wheelchair driving experience, have no known cognitive disability, and have no history of serious motion sicknesses. Participants signed a written consent form prior to any data collection. The study was ethically approved by the Central Research Ethics Advisory Group of the University of Kent. All participants read and signed a consent form. A total of 22 participants (aged between 18 and 43 and identified themselves as female (n = 10) and male (n = 12)) took part in the study. This allowed for evenly splitting the participants into two groups: Gamified VR Training (Game VR) and Realistic/Non-Gamified VR Training (NoGame VR). To assess the impact of a specific VR training on real-life driving skills, it was chosen for participants to exclusively undergo one VR training (Game VR or NoGame VR). After completing the study, participants were entered into a draw to win one of three Amazon vouchers (GBP 25, GBP 15, or GBP 10) as a token of appreciation for taking the time to participate in the study.

### 3.2. Setup of the Study

**Figure 1:**
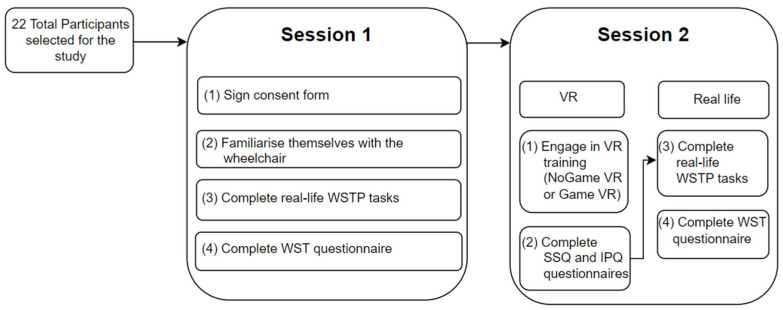
Flowchart of the study setup.

Participants completed two sessions in total. In the first session, participants were first introduced to the project aims, had the opportunity to ask questions, and were then asked to sign a consent form. Afterward, participants were given the opportunity to get acquainted with the real-life wheelchair, after which they completed a set of real-life driving tasks (see the following sections for details). At the end of the first session, participants filled out a WSTP-style questionnaire. After at least two days, but not more than a week, participants came back for their second session, in which they were randomly assigned either NoGame VR or Game VR training. During the VR training, the following parameters were collected: number of collisions, number of joystick movements (see Sum of Movement of the Joystick in Section 3.4.1), completion time for each task, and HR. The VR training was conducted up to three times (contingent upon the participants’ desire and experience of cybersickness), with a 5 to 10 min break in between. Where participants reported feeling unwell, the training was ceased immediately. After completing the VR training, the SSQ and the IPQ were administered. At the end of the second session, participants repeated the same set of real-life driving tasks as completed in their first session, followed by completing the WSTP Questionnaire. Figure 1 shows a flowchart of the setup of the study.

#### 3.2.1. Real-Life Environment for Skills Evaluation

**Figure 2:**
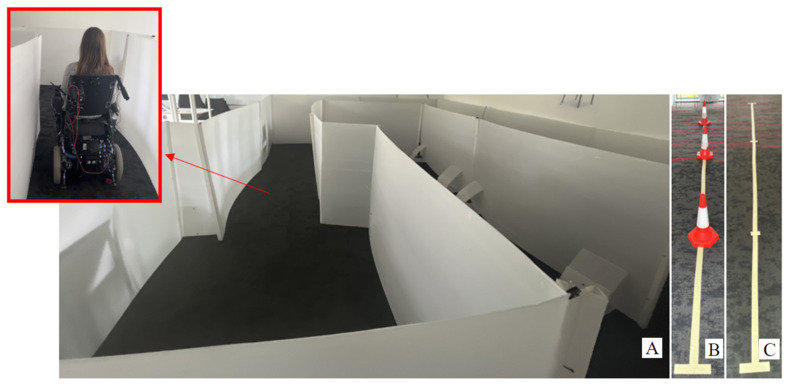
Real-life environment. Image (**A**) represents the room where the maze task was performed. Image (**B**) represents where the forward slalom and backward slalom tasks were performed. Image (**C**) represents where the forward and backward tasks were performed.

**Figure 3:**
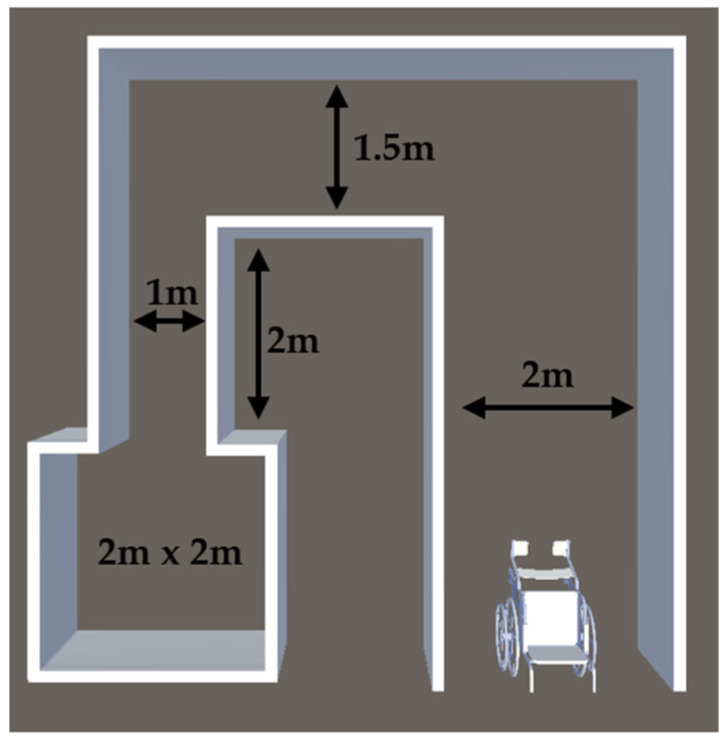
Real-life evaluation maze floorplan (not to scale).

The real-life tests were a replica of the tasks performed in VR. They consisted of driving forward and backward 10 m, driving through 3 obstacles placed 1.5 m apart both forward and back, and a maze. The setup was in a room as seen in Figure 2. The floor plan and size of the maze can be seen in Figure 3.

#### 3.2.2. NoGame VR Training

**Figure 4:**
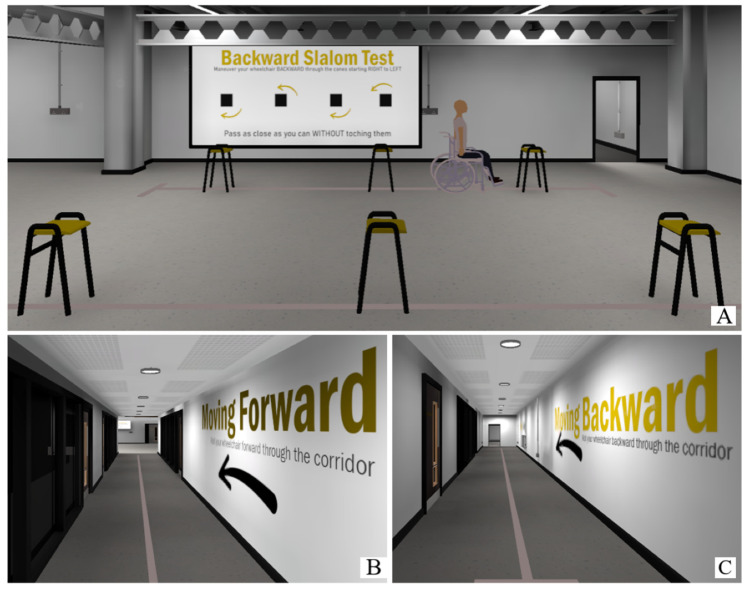
NoGame VR Training. Image (**A**) represents the room where the forward slalom test and backward slalom test were performed. Image (**B**) represents the room where the forward test was performed. Image (**C**) represents the room where the backward test was performed.

The realistic VR environment is a replica of part of the Jennison Building of the University of Kent (see Figure 4); in this scenario, participants performed the following 5 tasks (the same for Game VR): driving forward 10 m, driving backward 10 m, driving through 3 obstacles placed 1.5 m apart (both forward and backward), and driving through a maze. 

#### 3.2.3. Game VR Training

**Figure 5:**
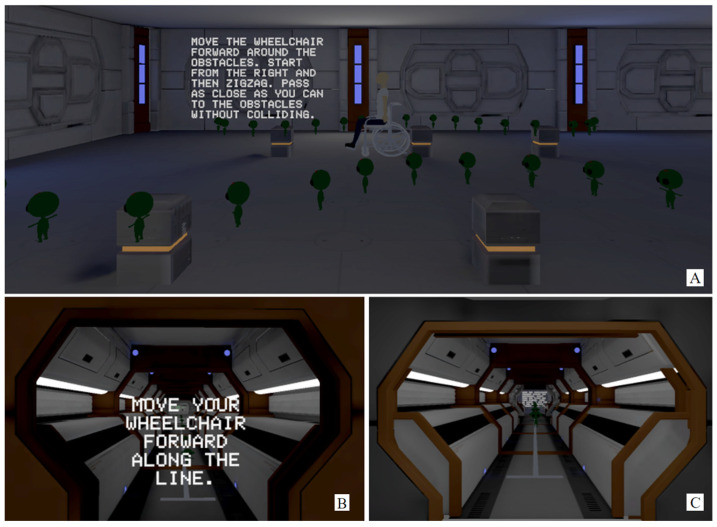
Game VR training. Image (**A**) represents the room where the forward slalom test and backward slalom test were performed. Image (**B**) represents the room where the forward test was performed. Image (**C**) represents the room where the backward test was performed.

**Figure 6:**
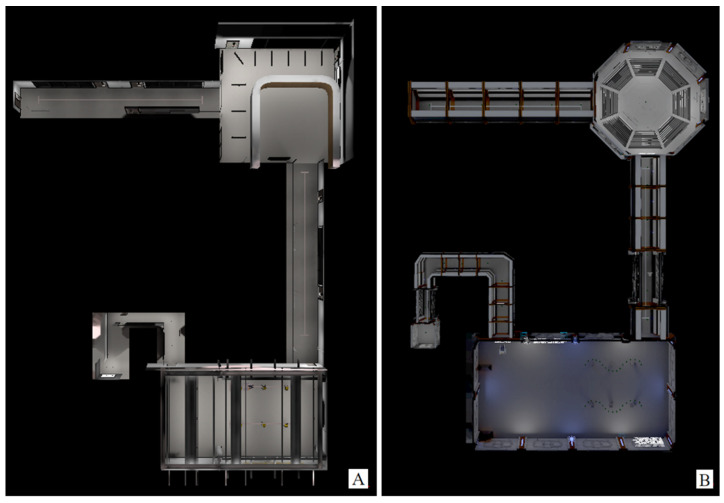
NoGame VR and Game VR training floorplans. Image (**A**) represents the floorplan view of the NoGame VR training, and image (**B**) represents the floorplan view of the Game VR training. The image is not to scale.

The gamified VR environment developed, as seen in Figure 5, is a non-realistic environment and contains the following 5 tasks (the same as for NoGame VR; see Figure 4): driving forward 10 m, driving backward 10 m, driving through 3 obstacles placed 1.5 m apart (both forward and backward), and driving through a maze. The floor plan of the Game VR environment compared to the NoGame VR environment can be seen in Figure 6.

The elements of gamification derive from the framework based on the self-determination theory of human motivation defined by Aparicio et al. [31,38]. This framework analyses tasks for gamification, considering the psychological and social needs of the participants. It incorporates appropriate game mechanics and evaluates the effectiveness of gamification based on fun, playability, and improved results using a quality service model [31] (which outlines three main needs to be addressed: autonomy, competence, and relatedness). For our environment, we decided to focus on competence. Examples of competence are positive feedback, optimal challenge, progressive information, intuitive controls, points, levels, and leaderboards. We incorporate positive feedback, optimal challenges, and points. As such, the design of Game VR training is based on a space shuttle and has the following elements of gamification: a target collection system (where the user follows the aliens to complete the tasks and to get points), positive visual feedback upon task completion, space shuttle background sound, and audio feedback upon collision. 

### 3.3. Hardware of the System

The participants were immersed in the environment by wearing an HMD, namely the Oculus Quest 2 (with a display resolution of 1832 × 1920 per eye and a refresh rate of 72 Hz) connected to a Microsoft PC (specifically the ROG Zephyrus M16 PC running on Windows 11 with Intel i9-12900H processor, GeForce RTX 3080Ti NVIDIA GPU, and 32 GB DDR5 RAM). Although the Oculus Quest 2 can be used as a standalone device, it was more convenient to connect it to the Microsoft PC to be able to assist participants by allowing researchers to see their actions on a monitor during the training and to store data more easily. The virtual wheelchair was controlled by an MPU-9250 IMU sensor fitted to the user’s wheelchair joystick (Dx2-REM550/551), which communicated with the Microsoft PC via a Wi-Fi hotspot (as described in the Controller Section 3.3.1 below). A polar H10 chest strap was used to measure the participants’ HR during the VR training. 

#### 3.3.1. Controller

**Figure 7:**
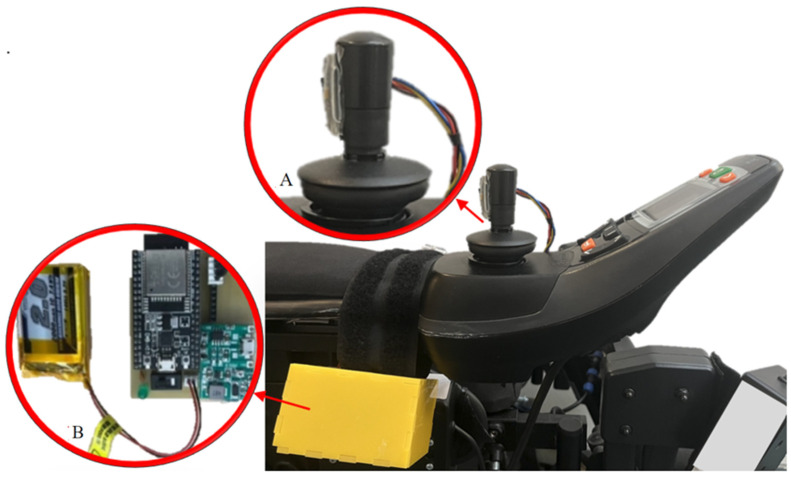
MPU-9250 sensor attached to the wheelchair’s joystick (Dx2-REM550/551) to control the VR System. Inset (**A**) shows the MPU-9250 sensor on the joystick. Inset (**B**) shows the ESP-32 microcontroller and the battery that powers it (housed in the yellow case), which is wired to the MPU-9250 sensor and wirelessly sends signals to the Microsoft PC.

The MPU-9250 (9-axis IMU, 3-axis magnetometer, 3-axis gyroscope, 3-axis accelerometer) sensor was connected to an ESP32 microcontroller and retrofitted on the wheelchair’s joystick (the Dx2-REM550/551) (see Figure 7). The microcontroller was fitted on a battery-powered PCB, thus making the controller system portable. The accelerometer values were collected by the microcontroller via an I2C interface and sent via a Wi-Fi hotspot to Unity 3D. The accelerometer was calibrated to determine the range of values corresponding to various angles related to movement commands, i.e., forward, backward, right, and left; these ranges were used by Unity 3D during navigation to control the position of the wheelchair in the VR environment. 

We also used the accelerometer as a measure of the improvement of driving skills in real life. See Section 3.4.1 for more details.

### 3.4. Data Collection and Processing

The number of collisions, completion time, and the total sum of movements of the joystick were collected using Unity3D, for both real-life evaluation and VR training. The HR was collected using the Polar Beats App [39]. The WSTP-type questionnaire, the SSQ, and the IPQ were filled out on paper by the participants. The number of collisions, completion time, and total sum of movements of the joystick were processed using MATLAB. All the collected data were stored, organised, and plotted via Microsoft Excel; once the data were organised in their final form, statistical analysis was performed using IBM SPSS. For all statistical tests, we use *p* = 0.05 as a standard of significance. The different parameters/data collected in this study are described in detail in the following subsections, first presenting the data collected to test H_1_, then H_2_, and finally H_3_. 

#### 3.4.1. Data Collected to Test H_1_: Real-Life Driving Performance after VR Training Will Differ Based on the Environment

##### Sum of Movement of the Joystick

The IMU sensor was placed on the joystick in the vertical direction (see Figure 7 extension A). The accelerometer values in the *x* and *y* directions (horizontal plane) were recorded for each task, both during the real-life sessions and the VR training, and used to calculate a proxy for the total sum of the movements of the joystick, *L*_jm_ for each task. Following Farago et al. [40], we applied the Pythagorean Theorem to the accelerometer data in the *x* and *y* plane to quantify a single movement of the joystick, and then we summed all those values to get a proxy of total movements. The joystick data for both the real-life sessions and VR training were collected using Unity3D. The real-life *L*_jm_ (from session 2 and for each task) from NoGame VR was compared with the one from Game VR through a Mann–Whitney U test; the same test was conducted for the *L*_jm_ from session 1 for those tasks that showed statistical significance, to check if the significant difference was truly the result of VR training.

##### Completion Time

The completion time, *C*_t_, for each task, was recorded both in VR and in real life using Unity3D. The real-life *C*_t_ (from session 2 and for each task) from NoGame VR was compared with the one from Game VR through a Mann–Whitney U test; the same test was conducted for the *C*_t_ from session 1 for those tasks that showed statistical significance, to check if the significant difference was truly the result of VR training.

##### Number of Collisions

The number of collisions, *N_c_*, with obstacles and the walls of the VR environment, was recorded both in VR and in real life using Unity3D. The real-life *N_c_* from session 2 of the two groups was compared through independent *t*-tests. If the *N_c_* showed statistical significance, then the real-life *N_c_* from session 1 was also tested to see whether the significant difference was a result of VR training.

##### WSTP-Style Questionnaire

The WSTP questionnaire was adapted by us to the tasks performed in our study; the original questionnaire has 27 questions [20] while the adapted one consists of 6 questions (see Appendix A). The adapted questionnaire was completed by the participants after each real-life session and asked them to perform a self-assessment of their driving skills. The scores from session 2 of the two groups were compared through independent *t*-tests. If any score showed statistical significance for any question, then the scores from session 1 of that question were also tested to see whether the significant difference was a result of VR training.

#### 3.4.2. Data Collected to test H_2_: The Levels of Involvement and Presence Will Vary Based on the Environment

##### Igroup Presence Questionnaire

The IPQ [21] asks the user to assign a score from 0 to 6 to 14 questions. Each question falls within one of the following categories: involvement, experienced realism, spatial presence, and general presence. Participants were given the questionnaire after VR training. The scores of each category were compared between the two groups with an independent *t*-test.

#### 3.4.3. Data Collected to Test H_3_: The Perceived Cybersickness Will Vary Based on the Environment

##### Heart Rate

The HR was measured using the Polar H10 sensor with the Pro Strap (as chest strap) [39]. The polar H10 uses ECG sensors and outputs HR in beats per minute (bpm) at a sampling rate of 1 Hz. The technical specifications of the sensor are given in Table 1. 

The HR measured throughout the whole VR training (and all tasks) for each participant was batched with the HR of the other participants from the same group; the two batches of HR data were compared using an independent *t*-test. As the test showed statistical significance, batches for each task from the two groups were compared with independent *t*-tests. 

##### Simulator Sickness Questionnaire

The SSQ [24] investigates perceived cybersickness by asking the user to score 16 symptoms from 0 to 3 (0—none, 1—slight, 2—moderate, 3—severe). This test was administered to the participants at the end of the VR training. Each of the symptoms corresponds to one or more of three categories: nausea, oculomotor, and disorientation. The scores for each category (N, O, D) and the total score (TS) were calculated using the following formulas [24]:

N = [a] × 9.54

O = [b] × 7.58

D = [c] × 13.92

TS = ([a] + [b] + [c]) × 3.74

As such, the total score of the symptoms in that category is reported. 

The SSQ results were analysed by calculating the average, SD, min, and max of each category (N, O, D) and the total score (TS). The scores N, O, D, and TS were then compared to reference scores [8] to quantify the level of cybersickness (i.e., none, slight, moderate, severe) caused by our system. The scores of each category were compared between the two groups with an independent *t*-test. 

## 4. Results

The collected data were processed using MATLAB and then analysed for statistical testing using IBM SPSS. Every data variable collected served to test a specific hypothesis. 

To test H_1_, we use the proxy of the total sum of joystick movements, *L*_jm_, and the completion time, *C_t_*, for each real-life task from session 2, to assess differences in real-life driving performances as a result of VR training between NoGame VR and Game VR, using independent-sample Mann–Whitney U tests as the dataset was small and did not follow normal distribution. For any task where a statistical difference was observed, we conducted independent-sample Mann–Whitney U tests for each corresponding real-life task from session 1, to check whether the statistical difference was the consequence of going through the VR training. For the WSTP-style questionnaire [17], we conducted independent *t*-tests. Independent *t*-tests were also carried out to analyse if there was any difference in the number of collisions, *N_c_*, in real-life driving performance. The results of these tests can be found in Section 4.1. 

To test H_2_, we conducted independent *t*-tests for the IPQ [21] results, as shown in Section 4.2. 

To test H_3_, we conducted independent *t*-tests for the HR and the SSQ [23], as reported in Section 4.3. 

The results are discussed in Section 5.

### 4.1. Results of the Data Collected to Test H_1:_ Real-Life Driving Performance after VR Training Will Differ Based on the Environment

#### 4.1.1. Proxy of the Sum of Joystick Movements, L_jm_

We used *L_jm_* to test for statistical significance between the two groups for the tasks conducted in real life in session 2 (after the VR training). We use an independent Mann–Whitney U test for the following null hypothesis:

**H_0_.**  *The distribution of both populations is identical.*

**Figure 8:**
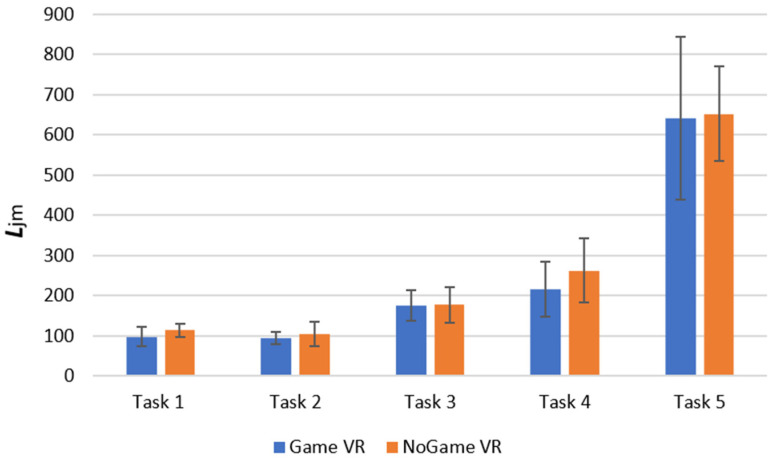
Session 2 real-life *L*_jm_ (proxy of the sum of joystick movement) for different tasks and groups. Task 1, forward; task 2, backward; task 3, slalom; task 4, backward slalom; task 5, maze.

We retain the null hypothesis for all tasks except for the forward task, where we rejected the null hypothesis (U = 81, *p* = 0.016), with the *L_jm_* for the group that trained in Game VR being statistically lower than the *L_jm_* for the group that trained in NoGame VR. Therefore, we conducted a Mann–Whitney U test for *L_jm_* for the forward task but this time from session 1, where we retained the null hypothesis as U = 61, *p* = 0.436. These findings suggest that, while participants from NoGame VR and Game VR exhibited similar performance for the forward task in session 1, a significant difference was present following the VR training, with the group that trained in Game VR demonstrating better performance. Further, the difference in performance in *L_jm_* between the groups is minimal, as seen in Figure 8. 

#### 4.1.2. Completion Time

We used *C_t_* to test for statistical significance between the two groups for the tasks conducted in real life in session 2 (after the VR training). We use this as an additional measure of difference in performance between groups, in conjunction with *L*_jm_. We use an independent Mann–Whitney U test for the following null hypothesis:

**H_0_.**  *The distribution of both populations is identical.*

We retain the null hypothesis for all tasks except for the forward task, where we reject the null hypothesis (*U* = 90.5, *p* = 0.010), with the *C_t_* for the group that trained in Game VR being statistically lower than the *C_t_* for the group that trained in NoGame VR. As such, we conduct a Mann–Whitney U test for *C_t_* for the forward task in session 1, where we retain the null hypothesis as *U* = 66, *p* = 0.230.

**Figure 9:**
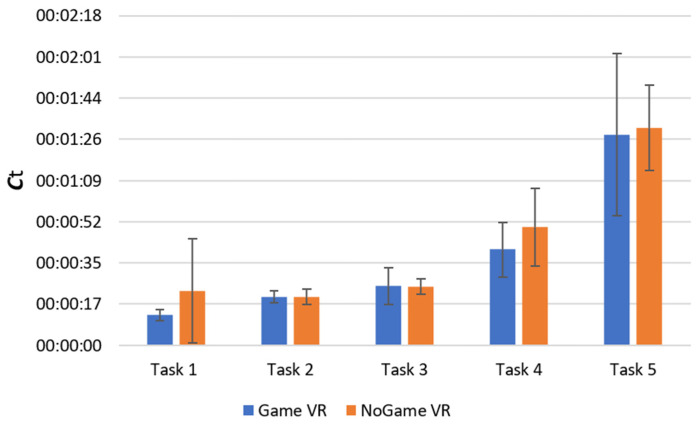
Session 2 real-life completion time. Figure legend: task 1, forward; task 2, backward; task 3, slalom; task 4, backward slalom; task 5, maze.

These findings indicate the reliability of the results obtained from Section 4.1.1, affirming that gamified VR training leads to improvements in the forward task in real life compared to non-gamified VR training. Furthermore, the performance in the completion time of most tasks is very similar between groups as seen in Figure 9.

#### 4.1.3. Number of Collisions

We compared the number of collisions, *N_c_*, of the real-life driving performances from session 2 between the two groups. We performed an independent *t*-test for the following hypothesis:

**H_0_.**  *(N_c_ mean over Game VR) − (N_c_ mean over NoGame VR) = 0*

The result of the test, *p* = 0.754, retained the null hypothesis, and therefore, there is no significant difference between the two groups.

#### 4.1.4. WSTP-Style Questionnaire

We used an adaption of the WSTP questionnaire as a further measure to determine the difference in real-life performance between the two groups in session 2. We performed independent *t*-tests for each question between the two groups and found no statistical difference.

### 4.2. Results of the Data Collected to Test H_2_: The Levels of Involvement and Presence Will Vary Based on the Environment

#### Igroup Presence Questionnaire

**Figure 10:**
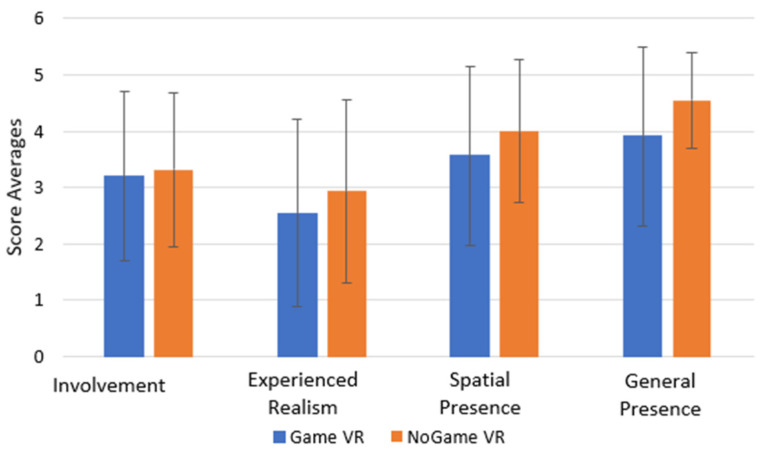
IPQ results.

The IPQ was administered after the VR training. Table 2 represents the results of the IPQ for the group that trained in NoGame VR, while Table 3 represents the results of the IPQ for the group that trained in Game VR. Figure 10 shows the results of both groups.

Surprisingly, NoGame VR demonstrated a higher overall perception of the different categories; although higher realism was to be expected for the non-gamified environment, the greater involvement and presence were unexpected. However, it is important to note that the standard deviation indicates a high variance in the results. Independent *t*-tests were conducted to analyse further these findings, which revealed no statistically significant difference. Consequently, while differences exist, they lack statistical significance.

### 4.3. Results of the Data Collected to Test H_3_: The Perceived Cybersickness Will Vary Based on the Environment

#### 4.3.1. Heart Rate

We compared the overall batched HR data between the two groups and the batched HR for each task between the two groups by performing independent *t*-tests with the following hypothesis:

**H_0_.**  *(HR mean over Game VR) − (HR mean over NoGame VR) = 0*

**Figure 11:**
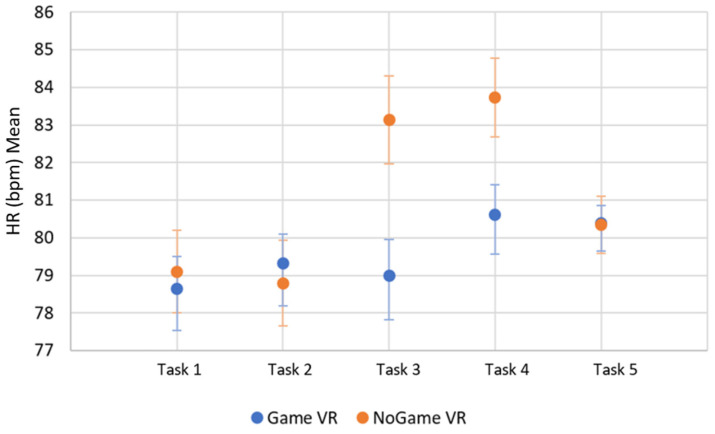
HR batched by task. The error bars represent the confidence interval.

The results for the overall HR rejected the null hypothesis, with the HR being statistically higher for the NoGame VR group with *p* < 0.001. In the tests for the individual tasks, the null hypothesis was retained for task 1—forward task, task 2—backward task, and task 5—maze task; therefore, there is no significant difference between the two groups for those tasks. However, for task 3—slalom task and task 4—backward slalom task, the results of the tests rejected the null hypothesis with *p* < 0.001 and *p* < 0.001, respectively; in these tests, the HR was higher for the NoGame VR group. The HR mean values and confidence intervals can be seen in Figure 11.

#### 4.3.2. Simulator Sickness Questionnaire

The SSQ was administered after the VR training for both groups. The results of NoGame VR are presented in Table 4, while the results of Game VR are presented in Table 5. We first compared the results to the reference scores in Table 6; then, we statistically compared the results between groups through an independent *t*-test.

Comparing the reference scores in Table 6 with the scores in Table 4 (NoGame VR), the non-gamified environment seems to provoke, on average, symptoms ranging between slight and moderate, with the total ranging between moderate and severe. The gamified environment, on the other hand, provokes, on average, symptoms ranging between none and slight, with the total ranging between slight and moderate. Because of these differences, we performed a one-sided independent *t*-test for the following hypothesis:

**H_0_.**  *(SSQ mean over Game VR) − (SSQ mean over NoGame VR) = 0*

The results show that the non-gamified environment seems to provoke significantly higher sickness symptoms for nausea (*t*_20_ = 3.332, *p* = 0.002), oculomotor (*t*_20_ = 1.857, *p* = 0.039), disorientation (*t*_20_ = 1.973, *p* = 0.031), and total (*t*_20_ = 2.575, *p* = 0.009). These results are very important in addressing our third hypothesis (**H_3_.** *The perceived cybersickness will vary based on the environment),* which in turn is key to answering our research question (*Can elements of gamification enhance the VR-based wheelchair training effectiveness?).*

## 5. Discussion

In this study, we aimed to address the research question *“Can elements of gamification enhance the VR-based wheelchair training effectiveness?*” which we investigated through three distinct hypotheses.

Our first hypothesis, “**H_1_** = *Driving performance after VR training will differ based on the environment*”, is not supported by our findings. The results indicate that the only task in which a significant difference in performance is observed after training, both in terms of *L_jm_* and *C_t_*, is the forward task. However, considering that this task does not require advanced skills to be completed and no significant differences are observed in other tasks, the statistical disparity may be attributed to chance. It is important to note that VR training was conducted during a single session/day and with the majority of participants undergoing VR training only once; our results may suggest that in order to observe a significant difference, more training sessions might be necessary.

Our second hypothesis, “**H_2_** = *The levels of immersion and presence will vary based on the environment*”, is also unsupported by our findings. Interestingly, participants in NoGame VR exhibited slightly higher average results in terms of the IPQ. However, the difference in results between the two groups is not statistically significant, and therefore, we cannot conclude that the non-gamified environment significantly elicits higher levels of presence. It is important to point out that Schwind et al. [42] also reported slightly higher scores in the IPQ for the non-gamified environment over the gamified one but with no significant difference. For more accurate IPQ results, Schwind et al. [42] suggested administering the IPQ during the VR experience rather than after it. Presence can be enhanced, instead, by adding more sophisticated haptic and visual feedback [43], as well as sound for directional cues to complete tasks [44].

Our third hypothesis, “**H_3_** = *The perceived cybersickness will vary based on the environment*”, is supported by our findings, which show a significant difference, as participants in NoGame VR experienced statistically higher symptoms of cybersickness compared to Game VR (according to the SSQ), as described in Section 4.3.2. Several studies suggested that reducing the field of view (FOV) can help alleviate cybersickness symptoms [45,46,47,48]. Although both groups had the same FOV, participants in Game VR had to collect targets (aliens), resulting in focusing their attention on a single object in their central vision. Our findings align with the study conducted by Yip and Saunders [49], who showed that directing the user’s attention to the central vision rather than peripheral vision has a positive impact on cybersickness. It is also possible that the presence of background noise contributed to the reduction in cybersickness symptoms, as found by Keshavarz and Hecht [50] who showed that pleasing sounds reduced cybersickness. Other studies [51], however, argue that sound has no effect on the level of perceived cybersickness.

Consistent with our findings, Nalivaiko et al. [52] found that participants with higher perceived cybersickness scores also showed an increase in HR. In our study, we found a statistically higher HR in participants of NoGame VR looking at the HR overall, which is the group with the higher SSQ scores. In terms of HR difference for specific tasks, NoGame VR had statistically higher HR values for the slalom task and backward slalom task, which may be the tasks in which participants experienced symptoms of sickness. Similarly, Salgado et al. [14] observed a correlation between increased HR and cybersickness, suggesting that HR increases as a result of cybersickness. Another physiological measure that is worthy of investigation in relation to cybersickness is electrodermal activity (EDA), as other studies believe changes in EDA are a result of cybersickness [53,54].

Finally, we found that the Game VR participants, who experienced less cybersickness, were more inclined to engage in repeated sessions of VR training. In fact, in Game VR, three participants repeated the VR training twice, and three repeated it three times. This could be attributed to the reduced incidence of cybersickness due to gamification, which consequently enhances the overall enjoyment and long-term sustainability of the VR experience. This is because cybersickness decreases enjoyment [55]; in fact, one participant stated, “Tasks in the reversed direction caused nausea. I had to stop working halfway”. In contrast, only two participants in NoGame VR attempted the training twice, but they had to stop the second attempt halfway due to severe nausea. These findings align with prior research conducted by Yildirim [55], who stated that feeling cybersickness decreases enjoyment. Garrido et al. [23] also found cybersickness to have a negative impact on enjoyment and future use of VR. Therefore, the implementation of a gamified environment in VR training holds the potential for broader adoption among the target population in need.

## 6. Conclusions and Future Work

This study aimed to examine the different effects of non-gamified versus gamified VR environments on the experience of wheelchair driving skills training, with the research question “*Can elements of gamification enhance the effectiveness of VR-based wheelchair training*?” To comprehensively investigate this research question, we considered three primary aspects: real-life driving skills performance, perceived presence and involvement, and cybersickness. Notably, no prior research has examined the impact of gamification on VR wheelchair driving skills training. Our findings do not provide conclusive evidence regarding the influence of gamification on the acquisition of real-life driving skills. In this study, participants undertook only one VR training session; therefore, multiple sessions (with more participants and different VR environments) are warranted in future investigations to test the long-term effect of gamification on wheelchair driving skills. In terms of perceived presence and involvement, no statistically significant differences were observed between the two environments. However, interesting results emerged in regard to cybersickness. Gamification was found to significantly reduce levels of perceived cybersickness and HR. Consequently, while users can obtain the appropriate wheelchair driving skills in VR training regardless of whether the environments are gamified or not, reducing cybersickness through gamification may enhance the usability and sustainability of the VR training by enabling users to repeat and enjoy the training as long as they need. As the difference in cybersickness between the two groups is significant, it would be interesting to explore how individual participants are affected by both VR environments.

## Figures and Tables

**Table 1:** Polar h10 specifications [39,41].

Battery Type	CR 2025
Battery Sealing Ring	O-ring 20.0 × 0.90 Material Silicone
Battery Lifetime	400 h
Sampling Rate	1 Hz
Operating Temperature	−10 °C to +50 °C/14 °F to 122 °F
Connector Material	ABS, ABS + GF, PC, Stainless steel
Strap Material	38% Polyamide, 29% Polyurethane, 20% Elastane, 13% Polyester, Silicone prints

**Table 2:** IPQ results for NoGame VR.

	Mean	SD
**Involvement**	3.32	1.36
**Experienced Realism**	2.93	1.63
**Spatial Presence**	4	1.27
**General Presence**	4.55	0.85

**Table 3:** IPQ results for Game VR.

	Mean	SD
**Involvement**	3.2	1.50
**Experienced Realism**	2.5	1.66
**Spatial Presence**	3.56	1.58
**General Presence**	3.91	1.58

**Table 4:** SSQ results for NoGame VR.

	Nausea	Oculomotor	Disorientation	Total
**Mean**	82.39	58.57	112.63	91.46
**SD**	24.24	35.24	56.18	37.45
**Min**	38.16	0	27.84	22.44
**Max**	114.48	113.7	208.8	145.86

**Table 5:** SSQ results for Game VR.

	Nausea	Oculomotor	Disorientation	Total
**Mean**	42.5	33.08	67.07	51
**SD**	31.46	28.82	52.02	36.23
**Min**	0	0	0	0
**Max**	95.4	83.38	139.2	97.24

**Table 6:** SSQ reference scores.

	Nausea	Oculomotor	Disorientation	Total
**None**	0	0	0	0
**Slight**	66.8	53.1	97.4	40.2
**Moderate**	133.6	106.1	194.9	80.4
**Severe**	200.3	159.2	292.3	120.5

## Data Availability

The data presented in this study are not publicly available due to participants’ privacy and ethics.

## Appendix A

User ID:	
**Adapted version of the Wheelchair Skills Test Questionnaire (WST-Q) Version 5.2 for Powered Wheelchairs**	
**Question**	**Answer**
How experienced are you in driving a wheelchair?	□ 1 □ 2 □ 3 □ 4 □ 5
How experienced are you with gaming?	□ 1 □ 2 □ 3 □ 4 □ 5
How experienced are you with VR?	□ 1 □ 2 □ 3 □ 4 □ 5
How realistic did the VR joystick feel?	□ 1 □ 2 □ 3 □ 4 □ 5
What is your age range?	□ 18–23 □ 24–28 □ 29–33 □ 34–38 □ 39–43 □ 44–48 □ 49–53 □ 54–58 □ 59–63 □ 63+
To which gender do you most identify?	□ Male □ Female □ Other: ……………… □ Prefer not say
**Introduction to the questionnaire**	
• Copies of this questionnaire can be downloaded from www.wheelchairskillsprogram.ca/eng/wstq.php (accessed on 25 December 2022).	
• More details about the questionnaire can be found there in the WSP Manual.	
• In this questionnaire, you will be asked questions about different skills that you might do in your wheelchair. These skills range from ones that are more basic at the beginning to those that are more advanced at the end.	
• There are no “right” or “wrong” answers. The purpose of the questionnaire is simply to help us understand how you use your wheelchair.	
• It will probably take about 10 min to complete the questionnaire, but please take as much time as you need.	
• If you have any comments, you will be able to record them at the end of the questionnaire.	
• For each specific skill, beginning on page 3. The questions and the possible answers are shown below.	

#	**Skill Description**		
1	Moving the wheelchair forward, for example along a hallway.	□ Yes, very well □ Yes, but not well □ Yes, with help □ No	□ Very confident □ Somewhat confident □ Somewhat unconfident □ Very unconfident
2	Moving the wheelchair backward, for example to back away from a table.	□ Yes, very well □ Yes, but not well □ Yes, with help □ No	□ Very confident □ Somewhat confident □ Somewhat unconfident □ Very unconfident
3	Turning the wheelchair around in a small space so that it is facing in the opposite direction.	□ Yes, very well □ Yes, but not well □ Yes, with help □ No	□ Very confident □ Somewhat confident □ Somewhat unconfident □ Very unconfident
4	Turning the wheelchair around obstacles while moving forward.	□ Yes, very well □ Yes, but not well □ Yes, with help □ No	□ Very confident □ Somewhat confident □ Somewhat unconfident □ Very unconfident
5	Turning the wheelchair around obstacles while moving backward.	□ Yes, very well □ Yes, but not well □ Yes, with help □ No	□ Very confident □ Somewhat confident □ Somewhat unconfident □ Very unconfident
6	Moving the wheelchair sideways in a small space, for example to get the side of your wheelchair next to a kitchen counter, and then back to where you started.	□ Yes, very well □ Yes, but not well □ Yes, with help □ No	□ Very confident □ Somewhat confident □ Somewhat unconfident □ Very unconfident

If you have any general comments about the questions that you have answered above, please record them in the space available below.

This is the end of the questionnaire. Thank you for completing it.
